# The influence of base pair tautomerism on single point mutations in aqueous DNA

**DOI:** 10.1098/rsfs.2019.0120

**Published:** 2020-10-16

**Authors:** A. Gheorghiu, P. V. Coveney, A. A. Arabi

**Affiliations:** 1 Centre for Computational Science, University College London, London, UK; 2 Informatics Institute, University of Amsterdam, Amsterdam, The Netherlands; 3 College of Medicine and Health Sciences, Biochemistry Department, United Arab Emirates University, PO Box 17666, Al Ain, United Arab Emirates

**Keywords:** high-performance computing, DNA mutation, proton transfer, multiscale modelling

## Abstract

The relationship between base pair hydrogen bond proton transfer and the rate of spontaneous single point mutations at ambient temperatures and pressures in aqueous DNA is investigated. By using an ensemble-based multiscale computational modelling method, statistically robust rates of proton transfer for the A:T and G:C base pairs within a solvated DNA dodecamer are calculated. Several different proton transfer pathways are observed within the same base pair. It is shown that, in G:C, the double proton transfer tautomer is preferred, while the single proton transfer process is favoured in A:T. The reported range of rate coefficients for double proton transfer is consistent with recent experimental data. Notwithstanding the approximately 1000 times more common presence of single proton transfer products from A:T, observationally there is bias towards G:C to A:T mutations in a wide range of living organisms. We infer that the double proton transfer reactions between G:C base pairs have a negligible contribution towards this bias for the following reasons: (i) the maximum half-life of the G*:C* tautomer is in the range of picoseconds, which is significantly smaller than the milliseconds it takes for DNA to unwind during replication, (ii) statistically, the majority of G*:C* tautomers revert back to their canonical forms through a barrierless process, and (iii) the thermodynamic instability of the tautomers with respect to the canonical base pairs. Through similar reasoning, we also deduce that proton transfer in the A:T base pair does not contribute to single point mutations in DNA.

## 1. Introduction

Mutations within DNA are crucial to both natural evolution and the occurrence of genetic diseases. Despite the protection of a cellular environment, exposure to various external agents such as free radicals, mutagenic compounds, electric fields, metallic centres or sources of radiation are known to cause mutations in DNA [1–3]. As part of their formulation of the DNA replication process, Watson & Crick [4] proposed that spontaneous mutations may arise as a consequence of base pair mismatching. These replication errors, known as *point mutations*, may naturally occur as a result of several types of mismatches such as wobble base pairing, Hoogsteen base pairing, ionization and tautomerism [5–7]. However, the frequency of each type of replication error is uncertain [8]. The purpose of the present paper is to investigate the kinetics and thermodynamics of base pair tautomerism in a realistic DNA model. The origin of these base pair tautomers is an ongoing subject of investigation that has already been studied by idealized gas phase quantum chemical models [9–19]. Despite this research, proton transfer between base pairs is still not completely understood.

**Figure 1.**
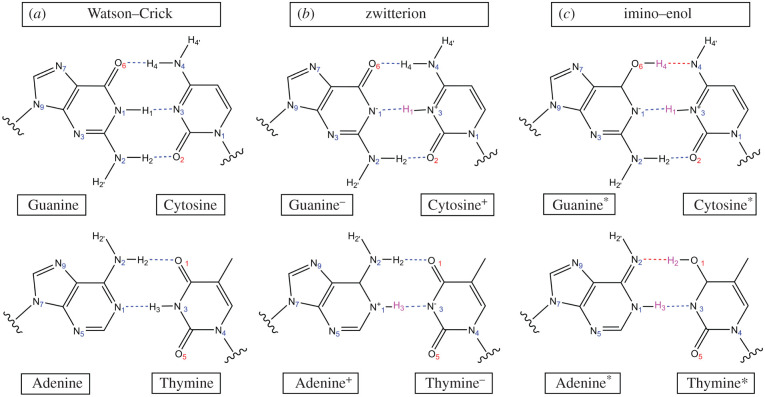
(*a*) Canonical Watson–Crick base pairs, (*b*) their single proton transfer zwitterion tautomers and (*c*) their double proton transfer tautomers (imino–enol). Transferred hydrogen atoms are highlighted in pink.

In 1963, Löwdin [20] sought out the biological implication of tautomerism as a consequence of proton tunnelling between DNA base pairs. Protons, obeying the laws of quantum theory, behave like wave packets. Therefore, owing to the quantum-mechanical (QM) tunnelling effect, there is always a small probability of proton transfer within the hydrogen-bonded network for all base pairs. Some of the products formed of base pair single and double proton transfer are shown in figure 1. Löwdin postulated that this transfer of protons over distances less than 1 Å might be the driving force for genetic mutations in all living organisms.

The proton transfers that occur as a result of hydrogen bonding can also be investigated as an inference of their acid–base chemistry. The p*K*_*a*_ values for the protonation of individual nucleobases in solution have been experimentally determined [21]. However, in the case of base pair hydrogen bonding within aqueous DNA, the ionization state is a function of the surrounding pH gradient [22]. In this study, neutral pH conditions are assumed throughout, which disfavours nucleobase ionization.

**Figure 2.**
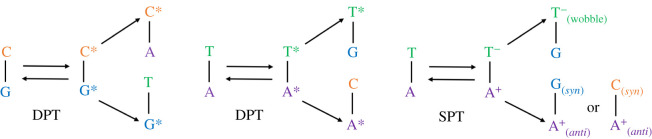
The types of base pair mismatches that may form during the DNA replication process as a consequence of double proton transfer (DPT) and single proton transfer (SPT).

In 1976, Topal & Fresco [23] introduced a more comprehensive set of base pairs (other than A:T and G:C) that are consistent with the geometric constraints of the standard double helix. These base pairs included both purine–pyrimidine (e.g. A:C and G:T) and purine–purine (e.g. A:A and G:A) mismatches. An example of how the purine–pyrimidine mismatches may form, as a consequence of base pair proton transfer, is shown in figure 2. A large majority of these base pair mismatches have been experimentally observed within aqueous DNA, in some cases within the active site of the DNA polymerase [6,24–29].

Recent density functional theory (DFT) calculations have shown that, within the approximation of implicit solvent, single proton transfer is thermodynamically a more favourable process than double proton transfer in the A:T base pair [30]. The single proton transfer A^+^:T^−^ zwitterion product facilitates mismatches in the form of wobble base pairing and base flipping (*anti-syn*), all of which do not fit within the restraints of the standard double helix [31–33]. The accurate replication of the Watson–Crick base pairs (G:C and A:T) is partly ensured by their high binding affinity to DNA polymerase [34]. There is a clear distinction in binding affinities to DNA polymerase for T*:G/G*:T and C*:A/A*:C with respect to all other base pair mismatches, which indicates that any other base pair mismatches are more likely to be flagged during the replication process for repair and, thus, not contribute to single point mutation rates [35].

The observed rates of spontaneous mutations must first be measured prior to determining the correlation between base pair tautomerism and spontaneous mutations. The rates of spontaneous mutations for DNA have been experimentally measured in a variety of living organisms, including humans [36,37]. The observed rates of spontaneous mutations in humans are low, estimated to be between $10^{-8}\text{ and }10^{-11}$ base pairs per nucleotide replication or up to 30 base pairs per genome [37–39]. In order to observe the effect of base pair tautomerism on the rate of spontaneous mutations, the equilibrium constant for the process must be greater than or equal to 10^−8^. This is due to the high-fidelity regulation of DNA repair during replication throughout the cell cycle [40,41].

This paper is divided into five further sections. Section 2 provides an introduction to previous proton transfer models and evaluates their shortcomings. Section 3 outlines the chemical pathways studied in this work and defines the physico-chemical parameters chosen in this study. Section 4 details the methods used to model DNA on the multiscale, bridging QM and molecular dynamics (MD) methods. The results are displayed in §5, followed by a discussion on the significance of base pair tautomerism for single point mutation rates in DNA. We draw our conclusions in §6.

## 2. Previous modelling of DNA

A:T and G:C dimers have been modelled with a solvent typically approximated by an implicit polarizable continuum model (PCM) [12,13,42]. One recent DFT study reported that the use of PCM was enough to change the mechanism of double proton transfer in the G:C base pair from concerted to stepwise [16]. Applying the PCM model is a computationally inexpensive way to approximate solvent, but does not capture the detailed local ordering of explicit water molecules around the reaction site of interest. Including realistic aqueous surroundings when modelling DNA is necessary to accurately describe base pair interactions and the proton transfer between them. Indeed, the main contribution to the stabilization of the DNA double helix does not come from inter-base pair hydrogen bonds, but rather from its interaction with surrounding solvent [43]. Within aqueous conditions, the cohesive base pair stacking interactions form a hydrophobic interior which maximizes the hydrogen bonding between base pairs. More recently, some authors have directed their attention towards multiscale modelling approaches to DNA, including in particular the application of QM/molecular mechanics (QM/MM) methods, in some instances further coupled to classical MD [10,44–52]. Several of these studies have investigated the effect of increasing the size of the QM region, including the insertion of explicit water molecules in the inner solvation sphere, the phosphate backbone or the adjacent base pairs [10,50–55]. Through the inclusion of several water molecules in the QM region, a ‘water-assisted’ base pair proton transfer pathway has been observed via Grotthuss-like transition states [50]. However, the activation energy associated with Grotthuss-like transition states is about twice that of direct proton transfer. Such mechanisms contribute to the rate process at higher order and, as we shall show, their effects lie within the errors of the dominant direct mechanism.

## 3. Description of physico-chemical parameters

This section displays the five fundamental chemical reactions underpinning base pair tautomerism, including their energetics, rates and equilibrium thermodynamics. These are as follows.

— The tautomerism of the G:C base pair via the concerted double proton transfer mechanism
  3.1 $$\text{G}:\text{C}\overset{k_{r}}{\underset{k_{r}^{\prime }}{\;⇌}}\text{G}{\;}^{*}:\text{C}{\;}^{*},$$
  whereby G*:C* is the double proton transfer tautomer; the forward and reverse rate coefficients are given by *k*_r_ and *k*′_r_ , respectively.
— The tautomerism of the G:C base pair via the stepwise double proton transfer mechanism
  3.2 $$\text{G}:\text{C}\overset{k_{a}}{\underset{k_{a}^{\prime }}{\;⇌}}{\left(\text{G}:\text{C}\right)}_{\mathrm{Int}}\overset{k_{b}}{\underset{k_{b}^{\prime }}{\;⇌}}\text{G}{\;}^{*}:\text{C}{\;}^{*},$$
  in which a two-step mechanism involving the formation of a single proton transfer intermediate ${\left(\text{G}:\text{C}\right)}_{\mathrm{Int}}$ is followed by the production of the G*:C* tautomer. The rate coefficients pertaining to the first and second steps are embellished by the subscripts ‘a’ and ‘b’, respectively.
— The single proton transfer in the G:C base pair via the concerted mechanism
  3.3 $$\text{G}:\text{C}\overset{k_{r}}{\underset{k_{r}^{\prime }}{\;⇌}}{\text{G}}^{-}:{\text{C}}^{+}$$
  whereby G^−^:C^+^ is the zwitterion product.
— The tautomerism of the A:T base pair via the concerted double proton transfer mechanism
  3.4 $$\text{A}:\text{T}\overset{k_{r}}{\underset{k_{r}^{\prime }}{\;⇌}}\text{A}{\;}^{*}:\text{T}{\;}^{*},$$
  where A*:T* is the tautomer product.
— The single proton transfer in the A:T base pair via the concerted mechanism
  3.5 $$\text{A}:\text{T}\overset{k_{r}}{\underset{k_{r}^{\prime }}{\;⇌}}{\text{A}}^{+}:{\text{T}}^{-},$$
  where A^+^:T^−^ is the zwitterion product.

The Gibbs free energy of the system is computed starting from the electronic energy, using DFT, with the inclusion of several thermal corrections

3.6 $$\Delta G=\Delta E_{\mathrm{corr}}+k_{B}T-\Delta S_{\mathrm{vib}}T,$$

where Δ*E*_corr_ is the vibrationally corrected electronic energy, *k*_B_ is the Boltzmann constant, *T* is the system temperature (300 K) and Δ*S*_vib_ is the vibrational entropic energy. The electronic energy corrected by quantized vibrations (*E*_corr_) is defined as

3.7 $$E_{\mathrm{corr}}=\epsilon_{0}+E_{\mathrm{vib}}+E_{\mathrm{ZPE}},$$

where *ε*_0_ is the electronic energy, *E*_vib_ is the vibrational energy term and *E*_ZPE_ is the zero point energy of the system.

According to conventional transition state theory, the rate coefficient of a first-order reaction *k* will have the following form [56]:

3.8 $$k(T)=\kappa (T)\frac{k_{B}T}{h}\text{exp}\left(-\frac{\Delta G^{‡}}{RT}\right),$$

where *T* is the temperature (300 K), *h* is Planck’s constant, $\Delta G^{‡}$ is the Gibbs free energy barrier and *R* is the universal gas constant. The tunnelling coefficient, *κ*(*T*), is given by the Wigner correction at second order [57],

3.9 $$\kappa (T)=1+\frac{1}{24}{\left(\beta \hbar \omega_{b}\right)}^{2}\;;\beta =\frac{1}{k_{B}T}\;,$$

where *ω*_*b*_ is the imaginary frequency of the transition state.

The equilibrium constant (K) for the reversible first-order reaction A $\overset{k_{r}}{\underset{k_{r}^{\prime }}{\;⇌}}$ B is expressed as

3.10 $$\text{K}=\frac{k_{r}}{k_{r}^{\prime }},$$

where *k*_r_ is the forward rate coefficient and *k*′_r_ is the reverse rate coefficient. The half-life (*t*_1/2_) of the species B is given by

3.11 $$t_{1/2}=\frac{\text{ln}2}{k_{r}^{\prime }}.$$

The rate coefficients for a reversible two-step reaction are defined as

3.12 $$A\overset{k_{a}}{\underset{k_{a}^{\prime }}{\;⇌}}I\overset{k_{b}}{\underset{k_{b}^{\prime }}{\;⇌}}P,$$

where A is the reactant, I is an intermediate and P is the product. The equilibrium constant (K) of this two-step reaction can be expressed as

3.13 $$\text{K}=\frac{k_{a}}{k_{a}^{\prime }}\times \frac{k_{b}}{k_{b}^{\prime }}={\text{K}}_{a}\times {\text{K}}_{b},$$

where K_a_ and K_b_ are the respective equilibrium constants for the first and second steps. In the case of *k*_a_ < < *k*^′^_a_, the two-step chemical reaction may be simplified to

3.14 $$A\overset{k_{a}^{\prime }}{\;\leftarrow }I\overset{k_{b}}{\underset{k_{b}^{\prime }}{\;⇌}}P.$$

Applying a steady-state approximation to the intermediate [I], the rate of the reverse reaction (the consumption of [P]) is expressed as

3.15 $$\frac{d[P]}{dt}=-\frac{k_{b}^{\prime }k_{a}^{\prime }}{k_{b}+k_{a}^{\prime }}[P].$$

Therefore, the overall reverse rate coefficient (*k*_r_′) for the multi-step reaction is approximated to

3.16 $$k_{r}^{\prime }=\frac{k_{b}^{\prime }k_{a}^{\prime }}{k_{b}+k_{a}^{\prime }}.$$

In equilibrium, the number of tautomeric base pairs per genome, *N*_taut_, is given by

3.17 $$N_{\mathrm{taut}}=\text{K}\times N,$$

where *N* is the size of the genome in base pairs and K is the double proton transfer equilibrium constant. To determine the number of zwitterion base pairs per human genome (*N*_zwitter_) at equilibrium, equation (3.17) is applied using the single proton transfer equilibrium constant.

In the case of G:C, the proportion of base pairs converted to tautomers after a certain time is given by the following equation describing the kinetics for a reversible first-order reaction [58]:

3.18 $${\left[{\text{G}}^{{\;}^{*}}{\text{C}}^{{\;}^{*}}\right]}_{t}=\frac{k_{r}{\left[\text{GC}\right]}_{0}-k_{r}^{\prime }{\left[{\text{G}}^{{\;}^{*}}{\text{C}}^{{\;}^{*}}\right]}_{0}}{k_{r}+k_{r}^{\prime }}\{1-\exp[-(k_{r}+k_{r}^{\prime })t]\},$$

where [G*C*]_*t*_ is the concentration of the rare tautomer at time *t* and *k*_*r*_ and *k*′_*r*_ are the forward and reverse rate coefficients, respectively. The concentrations $\left\{{\left[\text{GC}\right]}_{0},\;{\left[{\text{G}}^{*}{\text{C}}^{*}\right]}_{0}\;\text{and}\;{\left[{\text{G}}^{*}{\text{C}}^{*}\right]}_{t}\right\}$ in equation (3.18) are replaced by $\{{\left[\text{AT}\right]}_{0},\;{\left[{\text{A}}^{+}{\text{T}}^{-}\right]}_{0}\;\mathrm{and}$ ${\text{[}{\text{A}}^{+}{\text{T}}^{-}]}_{t}\}$ for the case of single proton transfer in the A:T base pair; the subscripts 0 denote the initial time.

## 4. Multiscale modelling of DNA

**Figure 3.**
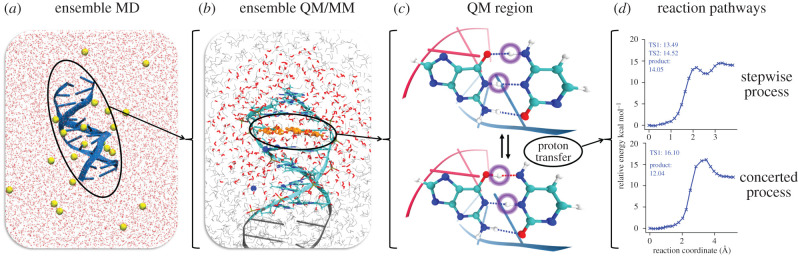
A schematic diagram displaying the multiscale workflow used in this work. (*a*) Ensemble-based classical molecular dynamics with the AMBER *parmbsc1* force field at 300 K and 1 atm is used to thermalize the DNA dodecamer (NAMD). (*b*) Ensemble-based QM/MM starting with initial configurations drawn from prior MD (ChemShell linking NWChem/DL-POLY). (*c*) The subsequent single base pair QM region (approx. 30 atoms). (*d*) The proton transfer reaction pathways from which the rate coefficients for the reaction are inferred.

Our model begins with the experimentally resolved structure of the B-DNA ‘Drew–Dickerson dodecamer’ d(CGCGAATTCGCG)_2_ [59]. Its conformational landscape is explored using ensemble-based classical MD, from which the configurations for the quantum chemical models are drawn. From there, an additional ensemble of quantum mechanics/molecular mechanics is performed to estimate the rates of base-pair proton transfer. A scheme showing the multiscale workflow used in this work is displayed in figure 3.

### 4.1. Ensemble-based classical molecular dynamics

The reproducibility of MD remains a topic of debate for many theoretical chemists and biologists [60–62]. Because of the extreme sensitivity to the initial conditions in any chaotic MD simulation, one-off simulations are not reliable. Instead, we can obtain statistically robust results by performing ensemble-based simulations, that is, a collection of *n* replicas each differing from the other solely in terms of the initial velocities assigned to all the atoms, drawn from a Maxwell–Boltzmann distribution at the temperature of interest [62]. Furthermore, ensemble-based simulations provide a reliable means of quantifying uncertainty in general [63].

The *parmbsc1* AMBER force field is used since it performs well for solvated double-helix DNA MD simulations [64,65]. The B-DNA structure of the Drew–Dickerson dodecamer (PDB ID. 1BNA) was neutralized (22 Na^+^) and solvated in a water box (dimensions: 71.15 Å × 73.13 Å × 85.94 Å) with the TIP3P water model [59,66]. Ensemble classical MD was then performed under periodic boundary conditions. The cut-off for the interaction distance for both electrostatic and van der Waals calculations was set to 10 Å. To prevent discontinuities in electrostatic and van der Waals energies, pairs of atoms greater than 11.5 Å apart were excluded. Periodic boundary electrostatics were calculated using the particle mesh Ewald method with a grid spacing of 1 Å. The following equilibration and simulation steps are repeated 10 times using the *parmbsc1* AMBER force field, generating a total of 100 ns simulation for the ensemble.

*Equilibration*: The coordinates of the entire DNA were restrained as the geometry of the rest of the system was minimized using the conjugate gradient and line search algorithm for 10 000 steps. Next, the temperature of the system was incrementally raised from 50 K to 300 K over a time period of 30 ps with a time step of 1 fs. The temperature of the system was maintained constant using a Berendsen barostat at a pressure of 1 atm. The restraints were then systematically removed over 0.5 ns, followed by an unrestrained 0.5 ns run.

*Simulation*: The production runs were for 10 ns, simulated at a constant temperature and pressure (300 K, 1 atm). All bonds between heavy atoms and hydrogen were constrained to their nominal length during integration and a time step of 2 fs was employed.

All MD simulations were performed using NAMD 2.12 on the UCL high-performance computing (HPC) facility *Grace*.

### 4.2. Choosing the quantum-mechanical method

A group of QM methods were compared on their ability to accurately describe base pair geometries and their interaction energies. Various QM methods that are known to accurately describe proton transfer barriers were chosen [67]. The QM methods assessed include B3LYP, CAM-B3LYP, LC-*ω*PBE, M06-2X and MP2, in conjunction with different basis sets and dispersion corrections. Both double zeta and triple zeta basis sets were assessed in conjunction with a double diffuse function to properly evaluate the energies of hydrogen bonds. To accurately capture weak dispersion interactions between base pairs, the Grimme-D3 correction and the exchange-hole dipole model (XDM) dispersion correction were individually assessed [68–70]. All QM calculations were performed using NWChem 6.6 on the Blue Waters supercomputer at the National Center for Supercomputing Applications, USA.^1^

**Figure 4.**
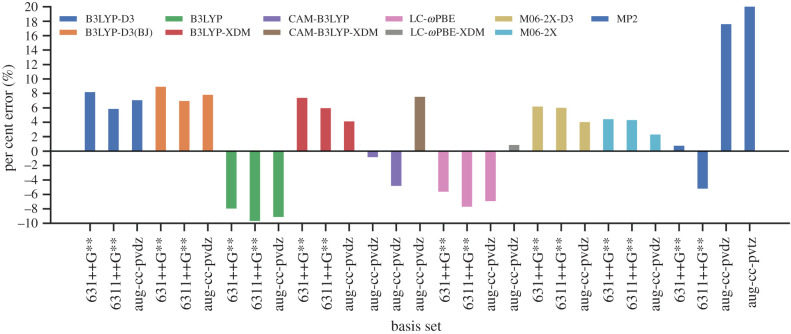
Per cent error in the binding energies of the hydrogen-bonded G:C base pair for a variety of selected QM methods and basis sets compared with the MP2(cc-pvtz)/CCSD(T)[CBS] reference value (−32.06 kcal mol^−1^) [71].

The geometry of a gas phase G:C base pair was optimized using each QM method and the guanine–cytosine interaction energies were calculated. The energies were then compared with highly accurate coupled-cluster values obtained by Hobza and co-workers [71] (as shown in figure 4). The RMSD of the selected base pair geometries were then compared with an MP2/aug-cc-pvdz optimized G:C base pair (further detailed in electronic supplementary material, S2). Although it is not feasible to use a triple zeta basis set, owing to its computational cost, both the double and triple zeta basis sets were considered in this benchmark for comparison purposes. In general, the triple zeta basis sets are expected to outperform the double zeta basis sets. However, in figure 4, it is shown that this is not the case for the Dunning basis sets in conjunction with the MP2 and CAM-B3LYP method. The same trend can be seen for the Pople basis sets, whereby 6-31++G** outperforms 6-311++G** when used with the B3LYP, LC-*ω*PBE and MP2 methods. This may be due to the cancellation of errors.

The interaction energies of stacked DNA base pairs were evaluated using B3LYP in combination with different basis sets and dispersion correction schemes. The same protocol as outlined in Šponer and co-workers [72] was followed and the interaction energies for 10 different combinations of stacked base pairs were calculated. A detailed definition of how the interaction energies are defined in addition to the computational methodology applied is offered in electronic supplementary material, S2.2. The results of the benchmark were then compared with the highly accurate DLPNO-CCSD(T)/CBS reference values (table 1). It is demonstrated that the B3LYP+XDM/aug-cc-pvdz binding energies compare well to the reference values for both the two-body and four-body stacking interaction energies. Despite its remarkable accuracy in predicting binding energies of hydrogen-bonded base pairs, CAM-B3LYP fails to accurately predict the binding energies of stacked base pairs.

**Table 1.** The computed average errors for the interaction energies of 10 combinations of stacked base pairs compared with DLPNO-CCSD(T)/CBS reference values [72]. The two-body and four-body stacking energies are given by Δ*E*_stack_ and Δ*E*_4stack_, respectively.

method	basis sets	average error in	average error in
		$\Delta E_{\mathrm{stack}}(\text{kcal}\;{\text{mol}}^{-1})$	$\Delta E_{4\mathrm{stack}}(\text{kcal}\;{\text{mol}}^{-1})$
B3LYP+D3	6-31++G**	−1.237	−2.000
	6-311++G**	−1.751	−2.462
	aug-cc-pvdz	−1.459	−2.144
B3LYP+D3(BJ)	6-31++G**	−1.889	−2.651
	6-311++G**	−2.402	−3.113
	aug-cc-pvdz	−2.111	−2.795
B3LYP+XDM	6-31++G**	−1.487	−1.867
	6-311++G**	−2.377	−2.520
	aug-cc-pvdz	0.782	0.345
CAM-B3LYP	6-31++G**	13.785	13.282
	6-311++G**	13.247	12.818
	aug-cc-pvdz	13.658	13.248

Therefore, the B3LYP/aug-cc-pvdz with XDM dispersion correction was selected as the QM method in this study, based on its ability to accurately reproduce the binding energies and geometries of both the hydrogen bonded and stacked base pairs (as shown in electronic supplementary material, S2).

### 4.3. Ensemble quantum mechanics/molecular mechanics

Previous base pair proton transfer studies that use *ab initio* MD are limited by the expense (and therefore the accuracy) of the QM method, by the size of the explicit base pair environment and by the accessible time scale of simulation [44,73,74]. The impracticality and cost of performing multiple *ab initio* MD simulations is obviated by the use of a multiscale ensemble based on the QM/MM method [75]. In this way, the statistical relevance of various proton transfer pathways can be assessed.

Despite numerous studies of DNA using QM/MM methods, there is no single agreed approach to modelling it [10,48–52]. Previous work has shown that the effects of adjacent base pair stacking can modify both the strengths and lengths of base pair hydrogen bonds, which ultimately influence the proton transfer energy profile [30,44,50]. However, a more recent QM/MM study (performed by Das *et al.* [52] in which 10 snapshots from a single MD simulation were studied) showed that adjacent base pair stacking has a relatively small influence on the base pair proton transfer energetic profile of the order of 1 kcal mol^−1^. The authors investigated the effect of neighbouring base pair polarization by systematically increasing the QM region size from one base pair through to five nucleosides. Of the 10 QM/MM calculations performed, the authors reported the electronic reaction energy for the single proton transfer in the G^+^:C base pair to vary between $7\text{ and }14\;\text{kcal}\;{\text{mol}}^{-1}$. The relatively large variance they observed (approx. ±3 kcal mol^−1^) indicates that the mean reaction energy is largely independent of (i) the QM region size and (ii) the polarization effects of the adjacent base pairs. In other words, the effect of using a larger QM region lies within the uncertainty of the single proton transfer itself.

We also independently considered the benefits that might accrue from increasing the QM region size to include the adjacent nucleotide base pairs. Our findings corroborate those of Das *et al.* In particular, while barrier heights from our one-off QM/MM calculations are lowered slightly, they lie within the errors arising from ensemble averaging of single base pair QM regions.

**Figure 5.**
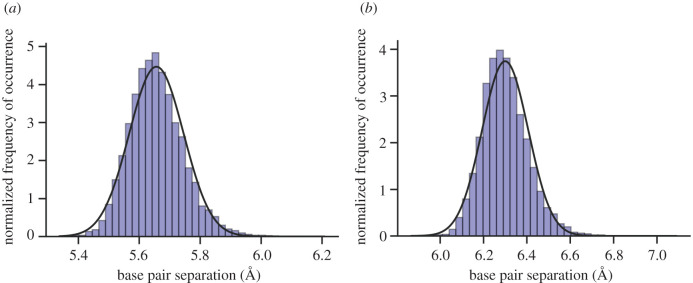
Histogram displaying the normal distribution of mean base pair distances—taken from a total of 100 ns DNA classical molecular dynamics simulation and 10 000 trajectory frames (detailed in §4.1). The continuous lines are the best fit Gaussian representative of the data. (*a*) G:C base pair (residues 3 and 22), (*b*) A:T base pair (residues 6 and 19).

The initial configurations for the subsequent QM/MM ensemble were selected from the prior classical MD simulations based on the distance between the base pairs (the distribution of these distances is shown in figure 5). By sampling from the average of this base pair distance distribution, *n* configurations are selected as starting points for the ensemble QM/MM study. The number of QM/MM replicas (*n*) is determined in §4.4.

The QM/MM simulations were performed using ChemShell 3.7 to link NWChem 6.6 (QM) with DL-POLY (MM). All of the QM/MM tasks and optimizations were performed using the DL-FIND module as implemented in ChemShell [76]. The periodicity of each initial QM/MM configuration was removed, which resulted in a solvation sphere of 15 Å containing approximately 9000 atoms. The QM region consisted of a single base pair with hydrogen linker atoms placed between the deoxyribose C1′ and the corresponding terminal N of the nucleobase. For this work, the electrostatic interactions between the QM and MM regions were modelled using electrostatic embedding. In principle, the use of a polarizable force field might be expected to provide a more realistic charge description at the price of introducing further fitting parameters. The use of such force fields in QM/MM calculations alters energetic calculations by up to *ca* 1 kcal mol^−1^ [77], while established electrostatic embedding techniques are more robust and computationally efficient than the best currently available counterparts [78]. In accordance with a previous ChemShell QM/MM DNA study [79], our ‘active’ MM region consists of all residues within 15 Å of the QM base pair; the remaining residues beyond this distance were frozen. This large active MM region was selected to permit structural changes in the phosphate backbone and the inner solvation sphere during geometry optimization. The proton transfer reaction pathways were calculated between the QM/MM optimized base pair and the proton transfer product using the adiabatic climbing image nudged elastic band (CI-NEB) method [80]. Depending on the curvature of the reaction pathway, the replica was then categorized as either a stepwise or concerted process. Transition states on the reaction pathway were further optimized using the dimer method [81] and verified by a single imaginary frequency in the Hessian. When appropriate, the geometry of the reaction pathway intermediate was optimized to a local minimum. The Hessian for the QM region of each geometry in the optimized proton transfer reaction pathway was then calculated to include thermal corrections of 300 K. Next, the rate coefficients for proton transfer were calculated using conventional transition state theory (equation (3.8)) as implemented in the ChemShell rate module [82]. All ChemShell QM/MM calculations were performed using the UK national supercomputer ARCHER.^2^

### 4.4. Uncertainty quantification

**Figure 6.**
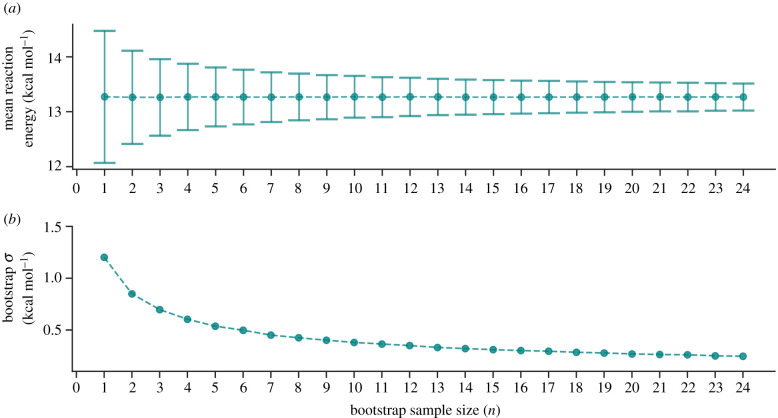
(*a*) The mean G:C → G*:C* tautomerism reaction energy (Δ*E*_rxn_) calculated using QM/MM (B3LYP+XDM/aug-cc-pvdz/AMBER). The error bars are the bootstrap standard deviation. (*b*) The bootstrap standard deviation (*σ*) of Δ*E*_rxn_, plotted against the number of QM/MM replicas *n*.

To establish an appropriate number of replicas *n* constituting the QM/MM ensemble, the bootstrap statistical method was employed [63]. By applying the QM/MM methodology as described in §4.3, the reaction energy (Δ*E*_rxn_) for the G:C → G*:C* tautomerism was calculated per replica. Figure 6 shows that the mean bootstrap standard deviation (*σ*) for the reaction energy (Δ*E*_rxn_) decreases as the number of replicas (*n*) increases. After 25 QM/MM replicas are included, the change in *σ* is negligible and, for this reason, *n* is set to 25.

## 5. Results and discussion

Our results show that double proton transfer tautomerism is the most frequent process in the G:C base pair, whereas the A:T base pair favours no reaction, with the subsidiary reaction being single proton transfer. The probability of occurrence for various proton transfer processes is summarized in table 2.

**Table 2.** The probability of occurrence of the various double proton transfer (DPT) and single proton transfer (SPT) mechanisms observed in the ensemble QM/MM study (sampled from 25 replicas per base pair).

	DPT		SPT		
base pair	concerted	stepwise	concerted	rearrangement	no proton transfer
G:C	0.12	0.84	0.04	0.00	0.00
A:T	0.00	0.00	0.28	0.04	0.68

While double proton transfer occurs 96% of the time in the G:C base pair, it was not observed at all in A:T. In fact, no reaction was observed for more than two-thirds (68%) of the A:T replicas. During the geometry optimization of the double proton transfer tautomer product (A*:T*), either one or two of the transferred protons were observed to revert back to their original nucleobases, which led to either no overall reaction or single proton transfer. The glaring instability of the A*:T* tautomer is a previously well agreed upon subject, whereby several researchers have instead reported on a metastable A^+^:T^−^ zwitterion intermediate and a highly unstable double proton transfer transition state [10,12,30,74]. In all our cases of single proton transfer in A:T, the same zwitterion product (A^+^:T^−^) was formed.

The double proton transfer process in G:C is split into two different types of pathways: the stepwise mechanism and the concerted mechanism. The probabilities of proton transfer pathways in G:C (as defined in table 2) show that the stepwise process occurs seven times more frequently than the concerted mechanism. Despite the tight restrictions imposed on average base pair distance (obtained from figure 5), the base pair rotation and torsion angles varied significantly between QM/MM replicas. As a consequence of this variation in configurations, many different proton transfer pathways were observed. In particular, one uncommon G:C QM/MM replica displayed the concerted single proton transfer process, while another in A:T (labelled as ‘rearrangement’ in table 2) involved an intra-adenine proton rearrangement. The statistical significance of these pathways is low and can only be thoroughly assessed by a much larger ensemble simulation. Therefore, these rarer cases of proton transfer are excluded from the following analysis, although they do highlight the delicate complexities of the proton transfer reaction pathway overall.

Details of each individual CI-NEB reaction pathway for each replica in the QM/MM ensemble can be found in electronic supplementary material, S3 and S4. The energetics, thermodynamics and kinetics for the three most frequent proton transfer mechanisms in G:C and A:T are shown in table 3.

**Table 3.** The energies, thermodynamics and rate coefficients for proton transfer reactions in the G:C and A:T base pairs. For the stepwise process, a and b denote the first and second steps, respectively. The electronic energy barriers are denoted by $\Delta E_{}^{‡}$ and reaction energy by Δ*E*_rxn_. The Gibbs free energy barriers are denoted by $\Delta G_{}^{‡}$ and the reaction Gibbs energy by Δ*G*_rxn_. The forward and reverse rate coefficients of the reaction are given by *k*_r_ and *k*′_r_, respectively. The equilibrium constant of the reaction is defined as K. Mean energies are calculated from the QM/MM ensemble using the B3LYP+XDM/aug-cc-pvdz/AMBER method. The standard deviation is denoted by *σ*. DPT, double proton transfer; SPT, single proton transfer.

	G:C stepwise DPT		G:C concerted DPT		A:T concerted SPT	
	mean	*σ*	mean	*σ*	mean	*σ*
relative electronic energies (kcal mol^−1^)						
$\Delta E_{}^{‡}$	—	—	15.94	2.13	7.13	2.03
$\Delta E_{a}^{‡}$	13.98	1.12	—	—	—	—
Δ*E*_Int_	13.35	1.18	—	—	—	—
$\Delta E_{b}^{‡}$	1.53	0.73	—	—	—	—
Δ*E*_rxn_	13.49	1.11	11.72	0.52	6.03	1.92
relative Gibbs free energies (kcal mol^−1^)						
$\Delta G_{}^{‡}$	—	—	12.46	1.86	4.40	2.25
$\Delta G_{a}^{‡}$	10.38	1.11	—	—	—	—
Δ*G*_Int_	11.41	1.07	—	—	—	—
$\Delta G_{b}^{‡}$	0.09	0.64	—	—	—	—
Δ*G*_rxn_	12.37	1.31	11.44	0.61	5.49	2.03
rate coefficient (s^−1^)						
*k*_r_	—	—	1.19 × 10^5^	1.98 × 10^5^	8.13 × 10^10^	9.36 × 10^10^
*k*′_r_	7.75 × 10^13^ ${\;}^{,a}$	6.68 × 10^13^	7.80 × 10^12^	9.14 × 10^12^	1.41 × 10^14^	5.55 × 10^13^
*k*_a_	2.06 × 10^6^	3.38 × 10^6^	—	—	—	—
*k*′_a_	1.17 × 10^14^	4.60 × 10^13^	—	—	—	—
*k*_b_	1.72 × 10^13^	1.27 × 10^13^	—	—	—	—
*k*′_b_	8.61 × 10^13^	7.51 × 10^13^	—	—	—	—
K	5.55 × 10^−9^	9.21 × 10^−9^	7.99 × 10^−9^	8.09 × 10^−9^	6.29 × 10^−4^	5.55 × 10^−4^
K_a_	1.55 × 10^−8^	2.16 × 10^−8^	—	—	—	—
K_b_	0.61	0.88	—	—	—	—
half-life, *t*_1/2_ (s)						
G*:C*	2.37 × 10^−14^	2.53 × 10^−14^	3.06 × 10^−12^	5.12 × 10^−12^	—	—
A^+^:T^−^	—	—	—	—	6.27 × 10^−15^	3.96 × 10^−15^

^a^Calculated using equation (3.16).

### 5.1. G:C tautomerism

**Figure 7.**
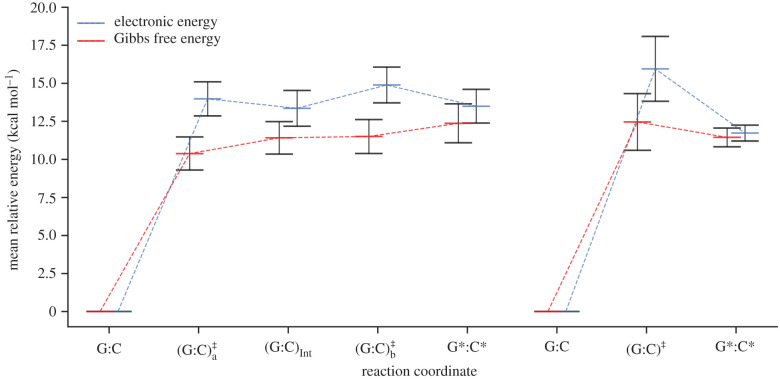
The electronic energy (blue) and the Gibbs free energy (red) as a function of the reaction coordinate for the stepwise (left) and concerted (right) double proton transfer in the G:C base pair. Energies are calculated relative to the energy of the reactant. Error bars are the standard deviations of the mean values.

The stepwise process is the most probable double proton transfer mechanism in G:C, occurring in 84% of the replicas from the QM/MM ensemble. This process proceeds via two transition states [(G:C)${\;}_{a}^{‡}$, (G:C)${\;}_{b}^{‡}$] and an intermediate (G:C)_Int_. The less frequent double proton transfer mechanism in G:C is concerted, occurring only 12% of the time. This process proceeds via a single transition state (G:C)^‡^ and has a larger electronic energy barrier of approximately 2 kcal mol^−1^ compared with the stepwise process. The optimized double proton transfer reaction pathways are displayed in figure 7. Tolosa *et al.* [83] were among the rare groups to report more than one possible reaction pathway for the double proton transfer reactions in DNA base pairs. They modelled a microhydrated G:C base pair using M06-2X/6-311++G** and steered MD. They reported stepwise, concerted and water-assisted mechanisms. The forward barrier height for the first step of the stepwise mechanism is 17.98 kcal mol^−1^, which is well out of the range we report. In addition, the barrier height they reported for the concerted mechanism was more than twice as high as our reported values.

The energetics, kinetics and thermodynamics for the stepwise double proton transfer mechanism are shown in the left column of table 3. The standard deviation for the mean electronic and Gibbs free energies are consistently approximately 1 kcal mol^−1^ for each step of the stepwise reaction coordinate. As shown in figure 7 (left), the energy of each part in the reaction coordinate lies within the standard deviation error of all the other points.

On the other hand, only three replicas from the QM/MM ensemble displayed the concerted G:C ⇌ G*:C* tautomerization pathway. The energetics, kinetics and thermodynamics for this pathway are shown in the middle column of table 3. Only three replicas participated in the concerted pathway, indicating the rarity of this phenomenon. As a result, the standard deviation of the energy barrier ($\Delta E^{‡}$) for the concerted process is twice that of the stepwise.

It is important to note that the overall shape of the double proton transfer reaction profiles, shown in figure 7, changes substantially between electronic and Gibbs free energy. The transition state energies are stabilized by approximately 3.5 kcal mol^−1^ in terms of the Gibbs free energy. In the case of the stepwise pathway (figure 7, left), this results in the transition states no longer being the maximum relative Gibbs free energies. Consequently, the overall instability of the G*:C* tautomer is reinforced as it occupies the highest Gibbs free energy state on the reaction coordinate. This is due to the zero-point energy contribution stabilizing both stepwise transition states ($\Delta G_{a}^{‡}$ and $\Delta G_{b}^{‡}$) by approximately 2 kcal mol^−1^ more than the intermediate and products. The computed barrier heights using B3LYP with a dispersion correction are expected to be slightly underestimated (although not to the extent of using B3LYP alone) [84]. The reverse barrier heights for the concerted mechanism in our study are 1.0 ± 1.5 kcal mol^−1^. These are in good agreement with the values of approximately 1 kcal mol^−1^ computed using BP86/6-311++G** [42]. Other studies, however, reported reverse free energy barriers less than 0 kcal mol^−1^ using MP2/aug-cc-pVTZ and B3LYP/6-311++G** [13,15].

**Figure 8.**
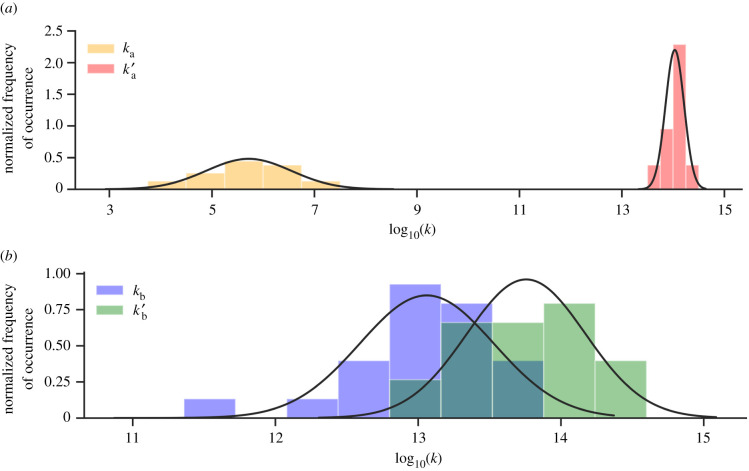
Histogram displaying the normal distribution of the log_10_(*k*) for the stepwise double proton transfer in G:C. (*a*) The forward *k*_a_ (orange) and reverse *k*′_a_ (red) rate coefficients of the first step and (*b*) the forward *k*_b_ (blue) and reverse *k*′_b_ (green) rate coefficients for the second step. The continuous lines show the best normal distribution fit for each dataset (sampled from 21 QM/MM replicas).

The range of the rate coefficients for each step in the G:C base pair stepwise double proton transfer is displayed as a normalized histogram in figure 8. The rate coefficients fit well to the normal distribution of the data, reinforcing the calculated means and standard deviations. The forward rate coefficient for the first step (*k*_a_), shown in orange, is approximately eight orders of magnitude lower than that of the reverse (*k*′_a_), shown in red. The reverse of the first step (G:C)_Int_ → G:C is favoured, establishing an equilibrium constant (K_a_) of the order of 10^−8^. The values for the forward (*k*_b_) and reverse (*k*^′^_b_) rate coefficients, coloured blue and green, respectively (figure 8*b*), are shown to partially overlap around 10^13^ s^−1^. The second step of this process (G:C)${\;}_{\mathrm{Int}}\;⇌\;$G*:C* lies close to equilibrium, with a K_b_ of approximately 0.6. By using equation (3.13), the overall stepwise double proton transfer equilibrium constant (K) is calculated to be of the order of 10^−9^. Recent computational studies have predicted the G:C double proton transfer equilibrium constant to be between $10^{-6}\text{\textbackslash{} }\mathrm{and}\;10^{-9}$ but this is largely dependent on the different QM approximations used [13–16,83,85]. Our results for K (approx. 10^−9^), are in agreement with Céron-Carrasco & Jacquemin (4.22 × 10^−9^) [85], who also used QM/MM methods to simulate proton transfer within a d(GGG)_2_ codon. They modelled the central base pair using M06-2X/6-311G**, the stacking base pairs using M06-2X/6-31G* and the sugar–phosphate backbone with the semi-empirical PM6 method. Despite predicting a similar equilibrium constant, they calculated the respective forward and reverse Gibbs free energy barrier to be 19.54 kcal mol^−1^ and 8.12 kcal mol^−1^, which are both approximately 8 kcal mol^−1^ larger than our results. Céron-Carrasco *et al.* [50] have shown that the inclusion of base pair stacking and explicit solvation causes the double proton transfer equilibrium constant to be smaller than that in the gas phase. In corroboration with their findings, we report equilibrium constants to be one order of magnitude smaller than recent QM-only gas phase studies that have used B3LYP/6-311++G** (1.68 × 10^−8^) and MP2/aug-cc-pvTZ (7.5 × 10^−8^) methods [13,15].

The rate coefficient of the first forward step (*k*_a_′) is the slowest by several orders of magnitude and, therefore, is the rate-determining step of the stepwise mechanism. For the stepwise process, the half-life of the G*:C* tautomer is calculated to be approximately 20 fs. The equilibrium constant (K) of the concerted mechanism is of the same order of magnitude as the stepwise mechanism (10^−9^). However, the reverse rate coefficient (*k*′_r_) is roughly two orders of magnitude smaller (approx. 10^12^ s^−1^) and, consequently, the half-life of the concerted G*:C* tautomer is calculated to be approximately 3 ps.

Previous gas phase base pair double proton transfer studies have typically observed only one of the G:C mechanisms shown in table 2 for a given QM method [10,12,13,17–19,30]. In doing so, the forward rate coefficient for the G:C double proton transfer has been estimated to be between $10^{2}\;\mathrm{and}\;10^{6}\;{\text{s}}^{-1}$, depending on the approximations made and the QM method used [14–16]. Because of the nature of these QM-only models, the associated errors in measuring the rate coefficient are not reported. A recent nuclear magnetic resonance (NMR) experimental study has estimated the lower bound for the rates of double proton transfer within the (G:T* ⇌ G*:T) enol tautomerism to be approximately 10^5^ s^−1^ or larger [33]. This G:T* base pair mismatch corresponds to guanine connected by three hydrogen bonds to a thymine enol tautomer and is comparatively similar to the G:C base pair. Similarly, the mean forward rate coefficients obtained from our ensemble QM/MM model are 10^6^ s^−1^ via the first step in the stepwise pathway (*k*_a_) and 10^5^ s^−1^ via the concerted pathway (*k*_r_). Our results are statistically robust as well as consistent with these experimental data, thus providing substantial improvements on the previous QM calculations.

### 5.2. A:T tautomerism

For the case of proton transfer occurring in A:T, the greatest percentage of QM/MM replicas (28%) display the concerted single proton transfer reaction. This concerted process involves the transfer of the proton in thymine to adenine to form the A^+^:T^−^ zwitterion (as shown in figure 1*b*). The preponderance of single proton transfer over double proton transfer is in agreement with a previous study, which demonstrated that the inclusion of explicit water molecules stabilizes the A^+^:T^−^ zwitterion over the A*:T* tautomer [42].

It should be noted that two of the QM/MM replicas performed have shown that the A:T → A*:T* double proton transfer reaction pathway can occur, although locating the exact transition states for these replicas was not possible. This is because the electronic energy levels of the approximate transition state and product (A*:T*) were within close proximity to each other, which resulted in an essentially flat reaction coordinate. Therefore, the transition state geometry optimization was unable to achieve satisfactory gradient convergence for the A*:T* tautomerism and the rate coefficients of the reaction were not determined. The A:T → A*:T* reaction coordinates are displayed in electronic supplementary material, S4. These two replicas were therefore removed from the QM/MM ensemble and replaced by another two, in accordance with the method described in §4.3.

**Figure 9.**
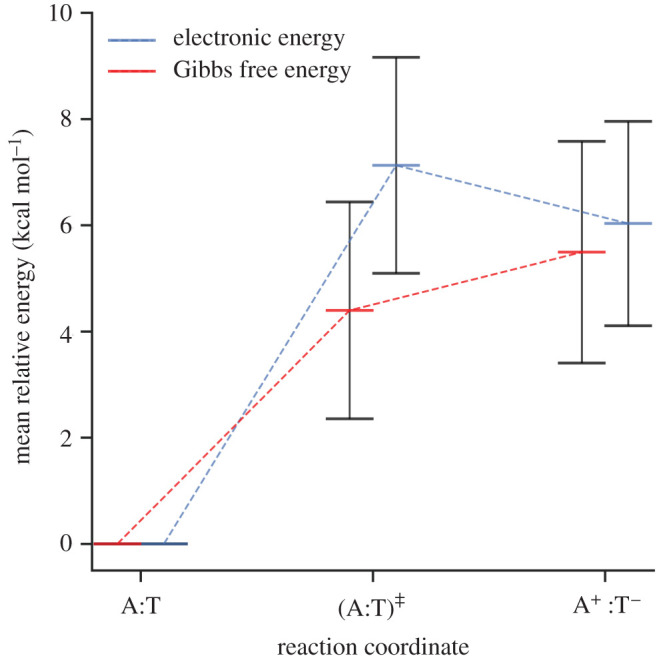
The Gibbs free energy (red) and electronic energy (blue) as a function of the reaction coordinate for the concerted single proton transfer in the A:T base pair. Energies are calculated relative to the energy of the reactant. Error bars are the standard deviations of the mean values.

The energetics, kinetics and equilibrium constants for the concerted single proton transfer (A:T → A^+^:T^−^) are presented in the right-hand column of table 3. The mean values and their standard deviations are calculated from a total of seven QM/MM replicas. The reaction coordinate for the concerted single proton transfer in A:T is shown in figure 9. The relative energies of the single proton transfer reaction in A:T (Δ*E*_rxn_, Δ*G*_rxn_) and the transition state ($\Delta E_{}^{‡}$, $\Delta G_{}^{‡}$) are within the standard deviation of one another, similar to that of G:C. The calculated values of the relative transition state energy ($\Delta E_{}^{‡}$) are unevenly distributed between 5.00 and 11.75 kcal mol^−1^, resulting in a large standard deviation (approx. 2 kcal mol^−1^). The Gibbs free energy modifies the single proton transfer reaction coordinate shape (figure 9), by stabilizing the transition state approximately 3 kcal mol^−1^ more than the A^+^:T^−^ zwitterion product (Δ*G*_rxn_). Several studies have calculated the reverse Gibbs free energy barrier to be 0.5 and 3 kcal mol^−1^ when using the M05-2X/6-311++G** and the M06-2X/6-311++G** methods, respectively, and in conjunction with the PCM solvent approximation [16,74]. Our work is partially in agreement with an earlier study by Céron-Carrasco *et al*. [30], who used the BP86/6-311++G** method and a micro-hydrated A:T model to predict the reverse free energy barrier to be approximately −1 kcal mol^−1^. However, the same study predicts the forward Gibbs free energy barrier to be approximately 2 kcal mol^−1^ smaller than ours (4.4 kcal mol^−1^). Since there is a distinct lack of QM/MM proton transfer models of A:T in the literature, we expect the difference in our results to be a consequence of the stacking and hydration effects we include. Overall, we find a negative reverse Gibbs free energy barrier for the single proton transfer reaction, which indicates that the A^+^:T^−^ zwitterion product is energetically unstable relative to the transition state (A:T)^‡^.

**Figure 10.**
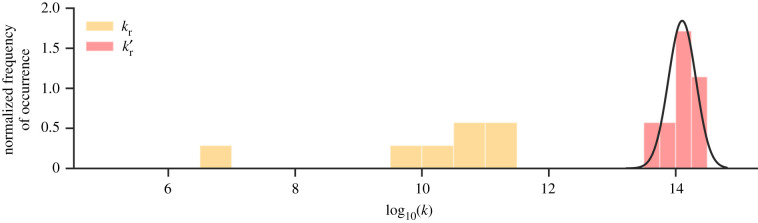
Histogram displaying the normal distribution of the log_10_(*k*) for the concerted single proton transfer in the A:T base pair based on seven replicas. The forward (*k*_r_) and reverse (*k*′_r_) rate coefficients are coloured yellow and red respectively. The continuous line shows the best normal distribution fit for the reverse rate coefficient (sampled from seven QM/MM replicas).

The distribution of the forward (*k*_r_) and reverse (*k*_r_′) rate coefficients for single proton transfer in the A:T base pair is displayed in figure 10. The distribution of *k*_r_ (yellow) has a much larger spread than *k*_r_′ (red). Because of this, the per cent standard deviation for the forward rate coefficient is much larger than that for the reverse reaction. The mean concerted single proton transfer rate coefficients *k*_r_ and *k*′_r_ are within the order of 10^10^ and 10^14^ s^−1^, respectively. The reverse rate coefficient is four orders of magnitude larger than the forward one, resulting in an equilibrium constant (K) for the single proton transfer reaction in A:T of the order of 10^−4^. From the reverse rate coefficient, the half-life of the A^+^:T^−^ zwitterion is estimated to be 6.3 ± 4.0 fs.

### 5.3. Relation between proton transfer and single point mutation rates

An upper bound on the spontaneous mutation rates in human DNA can be estimated under the following assumptions.

1. The size of the human genome (*N*) is 3 × 10^9^ bp and comprises purely a 50/50% G:C/A:T content.
2. The spontaneous mutations are exclusively a consequence of the proton transfer mechanisms shown in figure 2 (all other possible mutation pathways were neglected).
3. The single point mutation rates are estimated without consideration of post-replication DNA repair (e.g. proofreading mechanisms).
4. As a bare minimum, the Gibbs free energy of the tautomer must be lower than the Gibbs free energy of the transition state.
5. For the tautomer to contribute significantly to permanent mutations, the barrier associated with the reverse of proton transfer should be larger than approximately 3 kcal mol^−1^ [9].

If the above assumptions are satisfied, the proton transfer equilibrium constant (K) is proportional to the number of tautomers at equilibrium and, in turn, the maximum rate of spontaneous mutations per genome. Double proton transfer is assessed independently to single proton transfer, since the two mechanisms and their effect on spontaneous mutation rates *in vivo* are mutually exclusive.

The influence of the G:C double proton transfer reaction on spontaneous mutation rates is detailed in the left and centre columns in table 4. Despite occurring less frequently, the concerted pathway produces 33% more G*:C* tautomers at equilibrium than the stepwise pathway. It is estimated that there are a total of 20 G*:C* tautomers (*N*_taut_) within the human genome at equilibrium. It should be noted that the stepwise G*:C* tautomer product has a very short associated half-life (approx. 20 fs; table 3), as well as, on average, a barrierless reverse reaction (−1.0 ± 0.3 kcal mol^−1^ and $-0.8\pm 0.6\;\text{kcal}\;{\text{mol}}^{-1}$ for the respective first and second steps). Therefore, in accordance with assumption (4), the stepwise double proton transfer process in G:C is excluded from our single point mutation rate estimate. Given that the concerted tautomerism of G:C has a mean reverse Gibbs energy barrier of 1.0 ± 1.5 kcal mol^−1^, we are not able to say with certainty whether or not the reverse pathway is actually larger than 0 kcal mol^−1^, and thus there is no guarantee that the criterion of assumption (4) is met. Also, keeping in mind that the barrier heights in our study are slightly underestimated, as discussed above, and given that the 3 kcal mol^−1^ threshold in assumption (5) is only an approximation, we are not able to say with certainty whether or not assumption (5) is met. Therefore, we can neither assert that the probabilities of the mutations are null nor affirm that the concerted pathway leads to permanent mutations, especially since the time scale of DNA opening during replication is around one billion times larger [86] than the half-life of the G*:C* tautomer (approx. 3 ps). Therefore, it is reasonable to conclude that only a small fraction of the already small number of G*:C* tautomers (as a consequence of the concerted double proton transfer reaction) may lead to a permanent G:C → A:T mutation during DNA replication; in practical terms, it is truly negligible at less than one base pair per human genome replication.

**Table 4.** The equilibrium constant (K), the number of tautomer (*N*_taut_) or zwitterion (*N*_zwitter_) base pairs per human genome (no. bp) at equilibrium (estimated using equation (3.17)) and the per cent of proton transfer products formed after 1 s (estimated using equation (3.18)). A graphical representation of the tautomer concentrations can be found in electronic supplementary material, S5. DPT, double proton transfer; SPT single proton transfer.

	G:C stepwise DPT		G:C concerted DPT		A:T concerted SPT	
	mean	*σ*	mean	*σ*	mean	*σ*
K	5.55 × 10^−9^	9.21 × 10^−9^	7.99 × 10^−9^	8.09 × 10^−9^	6.29 × 10^−4^	5.55 × 10^−4^
*N*_taut_, bp	8	14	12	12	—	—
*N*_zwitter_, bp	—	—	—	—	9.44 × 10^5^	8.32 × 10^5^
% [G*C*] after 1 s	—	—	7.99 × 10^−7^	8.09 × 10^−7^	—	—
% [A^+^T^−^] after 1 s	—	—	—	—	6.29 × 10^−2^	5.54 × 10^−2^
time to reach equilibrium, s	—	—	1.70 × 10^−10^	2.30 × 10^−10^	3.39 × 10^−13^	2.00 × 10^−13^

By contrast, the formation of the double proton transfer A*:T* tautomer is rare and was not observed once within the entire QM/MM ensemble. On the other hand, the A:T → A^+^:T^−^ process was found to be significantly more thermodynamically and kinetically favourable than that of G:C → G*:C*. Specifically, there are on average five orders of magnitude more A^+^:T^−^ zwitterions than G*:C* tautomers at equilibrium, which contributes towards approximately 0.06% of the A:T content in the human genome. Despite this abundance of A^+^:T^−^ content, the zwitterions have a very short half-life (approx. 6 fs), as well as an average barrierless reverse reaction. Therefore, according to assumption (4), the zwitterion is not considered to influence mutation rates during DNA replication.

That G:C base pairs are more likely to spontaneously mutate is consistent with the universal G:C → A:T transition mutation bias observed *in vivo* during the replication process [39,87]. Despite the debate concerning the influence of external agents on this mutation bias, recent studies have reported that spontaneous mutations could be the driving force [88–90]. This bias is also observed in humans, whereby the mutation rate per genome replication for G:C sites is two orders of magnitude faster than for non-G:C sites [38,39]. Similar to recent studies by Brovarets & Hovorun [12,13], our work suggests that the double proton transfer tautomerism reactions in DNA are extremely unlikely to contribute towards the overall rates of transition mutations during the DNA replication process.

## 6. Conclusion

Through the use of ensemble QM/MM, multiple proton transfer pathways have been observed to occur within the canonical base pairs. We are the first to report a distribution of statistically robust rate coefficients for the most frequently occurring proton transfer mechanisms. The G:C proton transfer rate constants predicted in this paper are in better agreement with recent NMR experimental data than previous simulations [33]. The application of multiscale MD in conjunction with QM/MM enables us to sample realistic configurations for both the A:T and G:C base pairs within DNA under ambient conditions. Our results indicate that previous QM-only models may have oversimplified the processes that are involved in base pair proton transfer.

Both the stepwise and concerted double proton transfer pathways occur within the same G:C base pair. Despite the larger rate constants of the stepwise pathway, the calculated equilibrium constant (K) is similar to that of the concerted mechanism (approx. 10^−9^). As a consequence of both of the pathways, it is estimated that at equilibrium a total of 20 G*:C* tautomers are present in the human genome. However, the fast kinetics of the reverse reaction (G*:C* → G:C) promote the swift reverting of the rare tautomers to canonical G:C. Thus, there is only a negligible chance that the double proton transfer reaction can form G*:C* tautomers that last long enough to have any significant impact on the rates of point mutations in DNA, especially in the context of human genome replication.

On the other hand, A*:T* tautomerism is not observed in any QM/MM replicas within the ensemble. Indeed, the number of A*:T* tautomers in the human genome at equilibrium is then estimated to be negligible, compared with G*:C*. By contrast, we find that the A^+^:T^−^ zwitterion is 1000 times more likely to occur than the G*:C* tautomer in equilibrium. Despite its relative abundance, the A^+^:T^−^ zwitterion is not expected to cause base pair mismatches owing to its very short half-life (approx. 6 fs) and kinetic/thermodynamic instability.

## Supplementary Material

Supplementary Material: The influence of base pair tautomerism on single point mutations in aqueous DNA

## Endnotes

1 University of Illinois at Urbana-Champaign, URL: http://www.ncsa.illinois.edu/

2 EPCC at the University of Edinburgh, URL: https://www.epcc.ed.ac.uk/facilities/archer

## Data accessibility

The description of input data, structure and trajectory files can be found at https://github.com/gh3orghiu/.
