# Supplementary material for: The influence of base pair tautomerism on single point mutations in aqueous DNA
Source: Interface Focus. 2020 Oct 16;10(6):20190120. doi: 10.1098/rsfs.2019.0120 (PMC7653342; doi:10.1098/rsfs.2019.0120)
Supplement: Supplementary Material: The influence of base pair tautomerism on single point mutations in aqueous DNA [file rsfs20190120supp1.pdf]

Supplementary Material: The influence of base pair tautomerism on single point mutations in aqueous DNA

Gheorghiu A.[1], Coveney P. V.[1,2]*, Arabi A. A.[1,3]*

[1]*Centre for Computational Science, University College London, United Kingdom*
[2]*Informatics Institute, University of Amsterdam, Netherlands*
[3]*College of Medicine and Health Sciences, Biochemistry Department, United Arab Emirates University, PO Box 17666, Al Ain, United Arab Emirates*

September 23, 2020

1 Introduction

This document provides further information in support of the work presented in the main paper. The first section provides more detail about the quantum mechanical benchmarking of calculations on a gas-phase G:C base pair. The second section contains data obtained from the QM/MM ensembles, in particular reporting on results from the individual replicas whose overall statistical properties are described in the main article. The final section contains further information on 'rare' proton transfer pathways that have been observed within the ensemble-QM/MM method. The description of input data, structure and trajectory files can be found at `https://github.com/gh3orghiu/`.

2 Comparison of quantum mechanical methods

2.1 Guanine cyotsine hydrogen bonds

Figure S1 shows the mean absolute deviation of the displacement for all atoms in the G:C base pair compared to a reference structure that has been optimised using MP2/aug-cc-pvdz [S71].

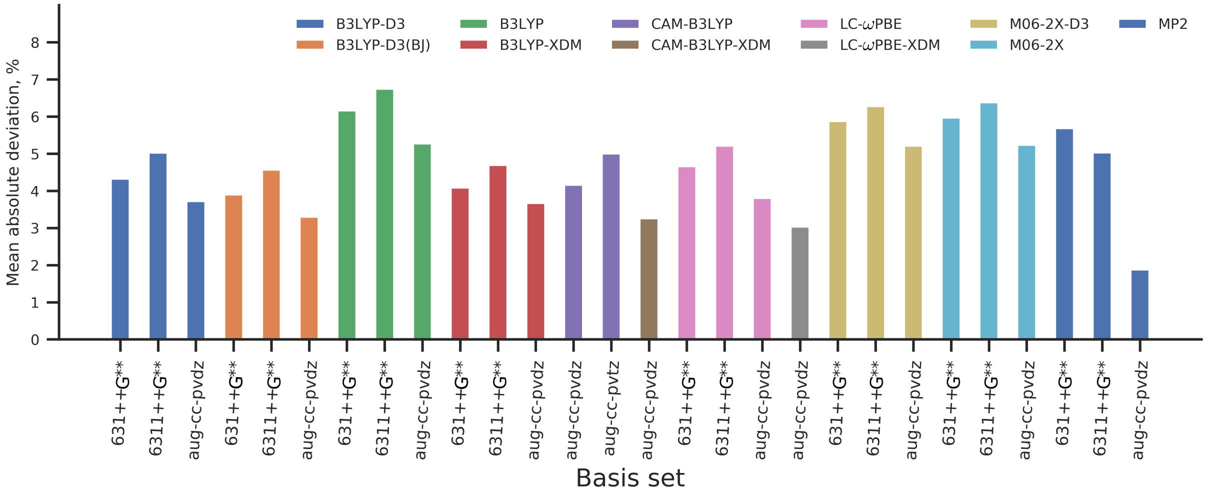

Figure S1: Percent mean absolute deviation (%) in the geometry coordinates of all atoms in a G:C base pair optimized with various DFT methods and basis sets compared to a G:C base pair optimized at the MP2/aug-cc-pvtz level [S71].

For all QM-methods the deviation of geometries to the benchmark G:C structure do not exceed 7%. The largest errors arise from the B3LYP functional (without dispersion correction) and the M06-2X functional. The MP2/aug-cc-pvdz has a very low deviation to the geometry because the structure we are comparing it to is also optimised using the MP2 method. The LC-ωPBE-XDM/aug-cc-pvdz method converges to both good base pair geometries and hydrogen bond interaction energies. The B3LYP-XDM/aug-cc-pvdz method was selected as our QM method since it represents the best trade-off between computational cost and accuracy.

2.2 Base pair stacking interactions

A variety of DFT methods were benchmarked on their ability to best describe the stacking interactions between two stacked base pairs. The stacking interactions of ten unique combinations of base pairs were calculated. An example of one stacked base pair combination (GC:CG) is shown in Figure S2).

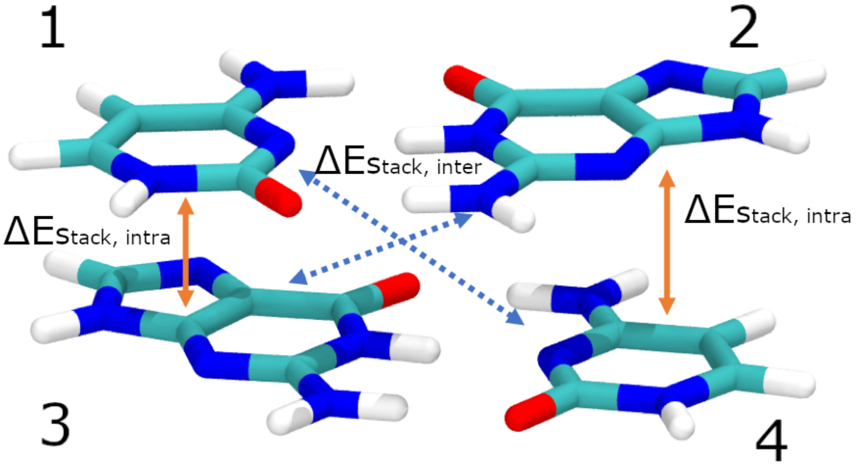

Figure S2: An exemplar stacked GC:CG base pair labelled with the different stacking interaction terms, where ΔE_{Inter} and ΔE_{Intra} are the inter and intra base pair interaction energies.

The initial geometries of the stacked base pairs and the reference interaction energies (calculated using CBS(T)-F12-CP) were taken from the Supplementary Material [S72]. From there, single point energies for each combination of stacked base pairs were performed using NWChem using a variety of QM methods. The two-body stacking energy for each base pair stack, ΔE_{stack} is calculated by the summation of the energies of the four-pair stacking interactions (as marked in Figure S2.)

$$\Delta E_{\text{stack}} = \Delta E_{13} + \Delta E_{24} + \Delta E_{14} + \Delta E_{23} \tag{S1}$$

where ΔE_{XY} is the interaction energy between nucleobases X and Y.

$$\Delta E_{XY} = E_{XY} - \sum_{i}^{X,Y} E_i \tag{S2}$$

The inclusion of the four-body interaction term is given by

$$\Delta E_{4\text{stack}} = E_{1234} - E_{12} - E_{34} \tag{S3}$$

3 Ensemble-QM/MM reaction pathways

The mean electronic energies for the three most commonly observed reaction pathways are displayed in Table S1. These are as follows: G:C step-wise double proton transfer; G:C concerted double proton transfer; and A:T concerted single proton transfer.

Table S1: Mean energies are calculated from the QM/MM ensemble using the B3LYP+XDM/aug-cc-pvdz/AMBER method; σ is the standard deviation.

	G:C Stepwise DPT		G:C Concerted DPT		A:T Concerted SPT	
	Mean	σ	Mean	σ	Mean	σ
Electronic Energies	(Hartrees)					
E_{Reactant}	-980.8587	1.1014	-979.3055	1.3884	-976.3413	8.3665
E_{TS}	—	—	-979.2801	1.3902	-976.3299	8.3654
E_{TSa}	-980.8364	1.1013	—	—	—	—
E_{Int}	-980.8374	1.1014	—	—	—	—
E_{TSb}	-980.8349	1.1013	—	—	—	—
E_{Product}	-980.8372	1.1015	-979.2868	1.3888	-976.3317	8.3659

3.1 G:C - Double proton transfer

The climbing image nudged elastic band (CI-NEB) pathways for each G:C replica in the 25-member QM/MM ensemble are shown in Figures S3 and S5. Each individual pathway is comprised of 24 images along the reaction coordinate. The electronic energies of the optimised geometries for the stepwise and concerted double proton transfer pathways are shown in Tables S2 and S3 respectively.

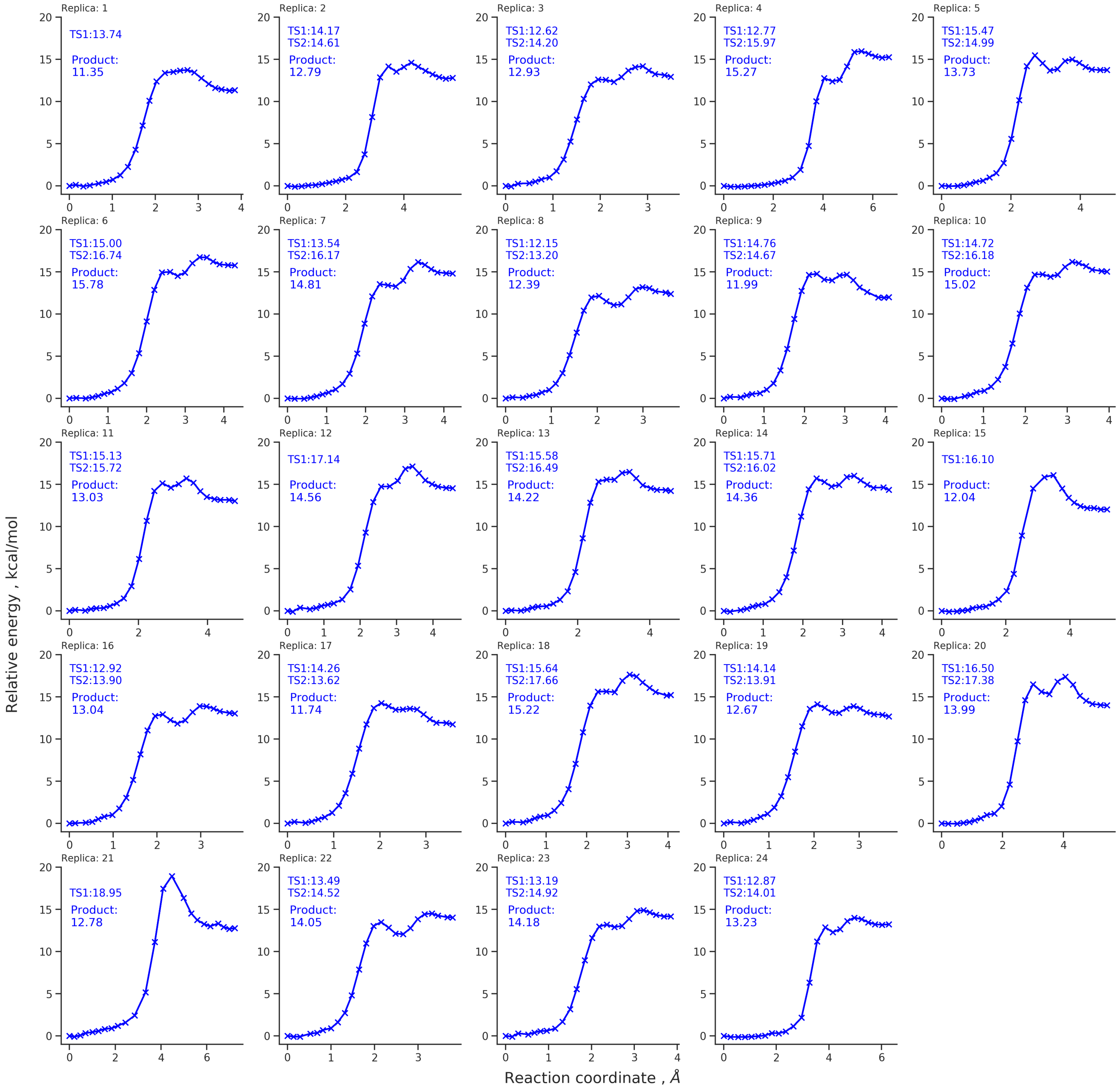

Figure S3: Climbing image nudged elastic band reaction coordinates for the double proton transfer G:C QM/MM-ensemble (24 replicas of the 25 total). Energies were calculated using the B3LYP+XDM/aug-cc-pvdz/AMBER method.

Concerted double proton transfer mechanisms occur in several replicas (1, 15 and 21), while the remaining replicas all occur via the stepwise mechanism.

Table S2: The QM/MM energy (Hartrees) for the stepwise double proton transfer in the G:C base pair ensemble. Each structure was optimised using B3LYP+XDM/aug-cc-pvdz/AMBER.

Replica	Reactant	TS1	Intermediate	TS2	Product
0	-980.566896	-980.544734	-980.545522	-980.544133	-980.546879
1	-981.390939	-981.370982	-981.371498	-981.368904	-981.370732
2	-981.161714	-981.141532	-981.141998	-981.136554	-981.137880
3	-979.764795	-979.741099	-979.743535	-979.741486	-979.743304
4	-979.965057	-979.941585	-979.942382	-979.938769	-979.940339
5	-980.704808	-980.683762	-980.684331	-980.679938	-980.681614
6	-980.262910	-980.244131	-980.245672	-980.242580	-980.243675
7	-981.593901	-981.570808	-981.572319	-981.571685	-981.575290
8	-979.628440	-979.604848	-979.605453	-979.602987	-979.604748
9	-981.722215	-981.698750	-981.699477	-981.698080	-981.701922
10	-981.099344	-981.075910	-981.076009	-981.073382	-981.076637
11	-980.205485	-980.180773	-980.181051	-980.180008	-980.183273
12	-982.092312	-982.068295	-982.069653	-982.067698	-982.069956
13	-981.696406	-981.676106	-981.677598	-981.674531	-981.676079
14	-980.788687	-980.766568	-980.767806	-980.767619	-980.770335
15	-982.902222	-982.877994	-982.878324	-982.875393	-982.878485
16	-981.222373	-981.200615	-981.201878	-981.200902	-981.202573
17	-980.153355	-980.127856	-980.129587	-980.127308	-980.131478
18	-982.965844	-982.944689	-982.946709	-982.942928	-982.943840
19	-978.206830	-978.186056	-978.186455	-978.183452	-978.184685
20	-979.937610	-979.917293	-979.917978	-979.915549	-979.916978

Table S3: The QM/MM energy (Hartrees) for the stepwise double proton transfer in G:C base pair ensemble. Each structure was optimised using B3LYP+XDM/aug-cc-pvdz/AMBER.

Replica	Reactant	TS1	Product
0	-979.078206	-979.056561	-979.060480
1	-981.108189	-981.083478	-981.089597
2	-977.730196	-977.700326	-977.710449

3.2 A:T - Single proton transfer

The climbing image nudged elastic band reaction coordinate for each single proton transfer A:T replica in the QM/MM ensemble is shown in Figure S4. Each individual pathway is comprised of 24 images along the reaction coordinate. The QM/MM energies of the optimised geometries for the concerted single proton transfer pathway are shown in Table S4.

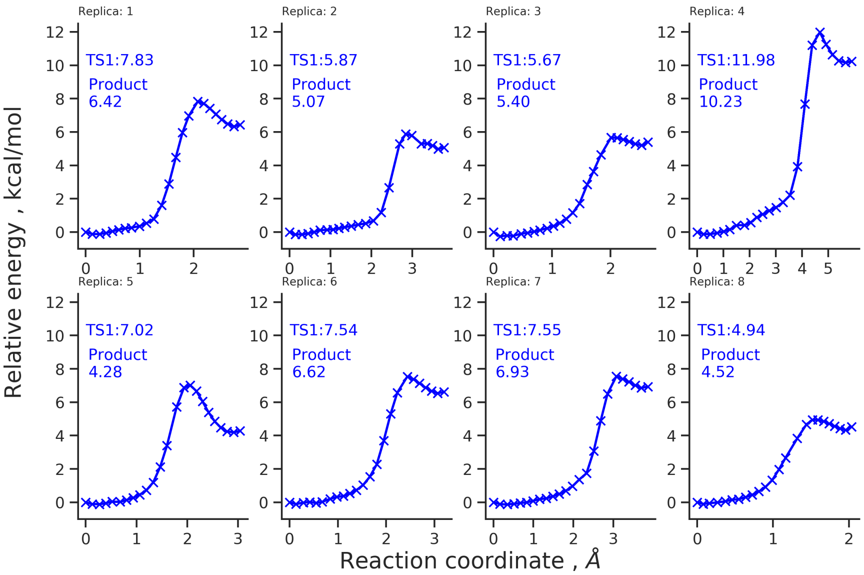

Figure S4: Climbing image nudged elastic band reaction coordinates for the seven A:T replicas that show single proton transfer - out of 25 replicas from the QM/MM-ensemble. Energies were calculated using the B3LYP+XDM/aug-cc-pvdz/AMBER method.

Table S4: The QM/MM energy (Hartrees) for the concerted single proton transfer in the A:T base pair ensemble. Each structure was optimised using B3LYP+XDM/aug-cc-pvdz/AMBER.

Repica	Reactant	TS1	Product
0	-982.101844	-982.090010	-982.091840
1	-981.738092	-981.728200	-981.729694
2	-982.791574	-982.782321	-982.783328
3	-981.030414	-981.011677	-981.013880
4	-980.275188	-980.264794	-980.268556
5	-964.739791	-964.728369	-964.729645
6	-961.712199	-961.704218	-961.704888

4 Rare cases of proton transfer

This section details the proton transfer mechanisms that we observed throughout the ensemble-QM/MM study. Due to the rarity and statistical insignificance of these processes, they are only discussed here for completeness.

4.1 G:C - Concerted single proton transfer

The QM/MM energies of the optimised geometries for the concerted single proton transfer pathway in G:C are shown in Table S5.

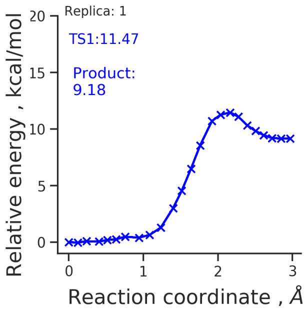

Figure S5: Climbing image nudged elastic band reaction coordinates for the singe proton transfer G:C QM/MM-ensemble (observed in 1 replica of the 25 total). Energies were calculated using the B3LYP+XDM/aug-cc-pvdz/AMBER method.

Table S5: The QM/MM energy (Hartrees) for the concerted single proton transfer in G:C base pair. Each structure was optimised using B3LYP+XDM/aug-cc-pvdz/AMBER.

Replica	Reactant	TS1	Product
0	-980.171642	-980.154563	-980.1574

4.2 A:T - Intra-rearrangement single proton transfer

One of the eight single proton transfer replicas for the A:T QM/MM-ensemble displayed a highly energetic intra-thymine rearrangement transition state, as shown in Figure S7.

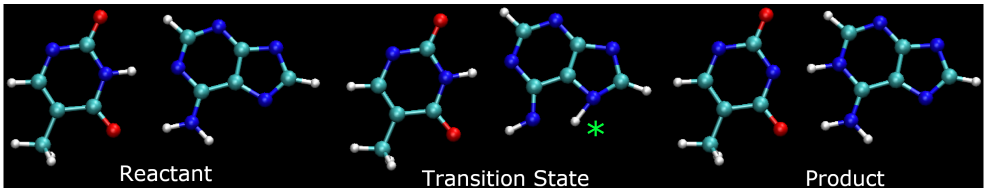

Figure S6: The mechanism for single proton transfer in A:T involving a high energy the intra-thymine rearrangement . The rearranging proton in this process is shown by the green asterisk.

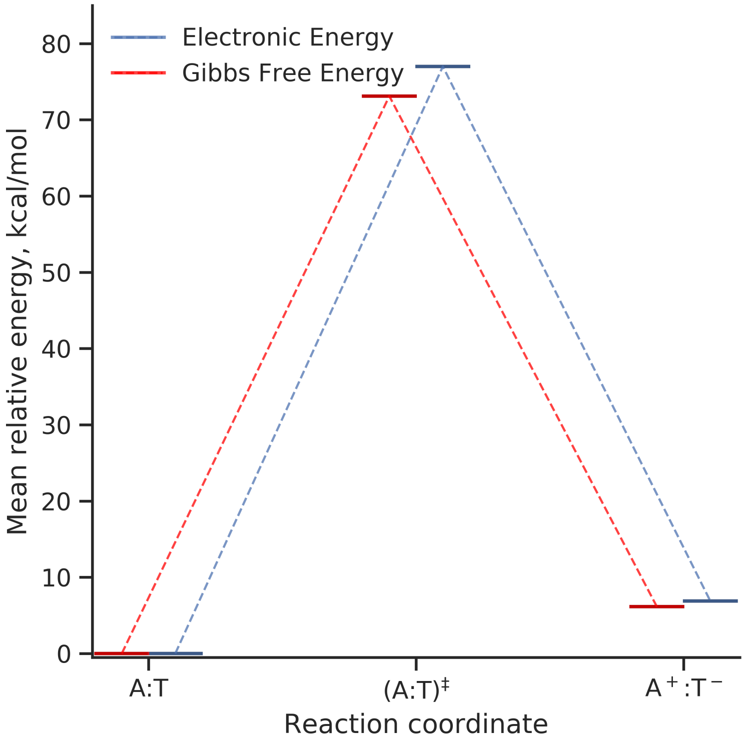

Figure S7: The electronic energy (blue) and the Gibbs free energy (red) as a function of the reaction coordinate for the intra-thymine rearrangement single proton transfer in the A:T base pair. Energies are calculated relative to the energy of the reactant.

Due to its extremely high activation energy of ∼70 kcal/mol, this transition state is deemed to be essentially irrelevant to the single proton transfer pathway.

4.3 A:T - Concerted double proton transfer

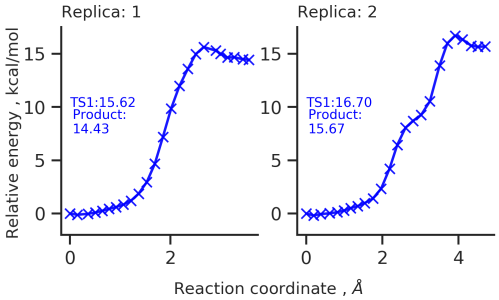

Figure S8: Climbing image nudged elastic band reaction coordinates for the A:T QM/MM-ensemble that show A:T→A*:T* double proton transfer. Energies were calculated using the B3LYP+XDM/aug-cc-pvdz/AMBER method.

Due to unconverged transition state geometries, the two replicas shown in Figure S8 that displayed double proton transfer within A:T were replaced by two additional replicas in the final A:T QM/MM ensemble.

5 Rare tautomer concentration at equilibrium

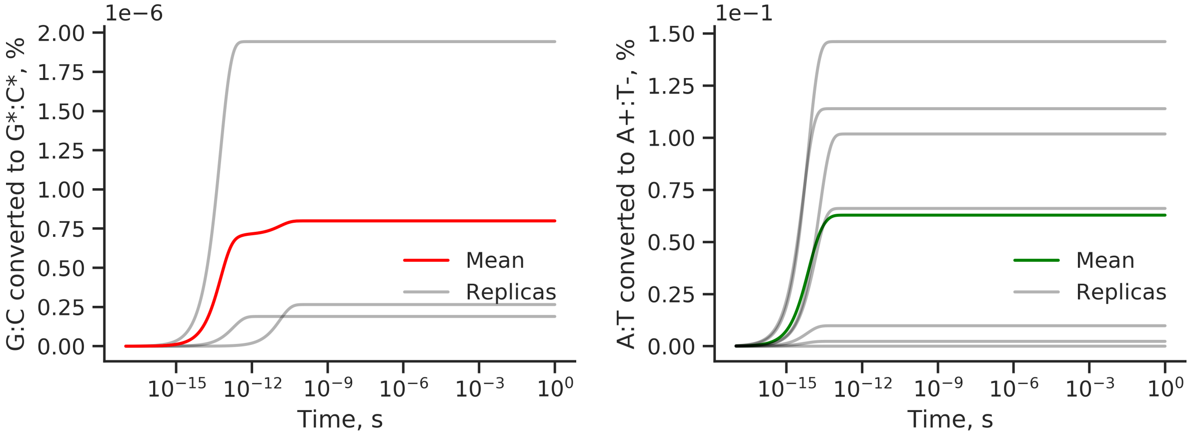

(a) G:C→G*:C*, Concerted

(b) A:T→A^+:T^-, Concerted

Figure S9: The percentage of canonical base pairs converted to proton transfer products over time, assuming the initial concentration of the proton transfer product is 0 - calculated using eq. (18). The mean values are represented by the bold red and green lines, while the individual values per QM/MM replica are represented by the light grey lines.

It takes approximately 10^{-10} seconds for the concentration of the G*:C* tautomer to reach equilibrium, which does not exceed the concentration of 8×10^{-7}%. The A^+:T^- zwitterion reaches equilibrium faster, at 10^{-13}s and at a significantly higher concentration ($6{\times}10^{-2}$ %) than G*:C*.
